# Enhancing hERG Risk Assessment with Interpretable Classificatory and Regression Models

**DOI:** 10.1021/acs.chemrestox.3c00400

**Published:** 2024-05-23

**Authors:** Igor H. Sanches, Rodolpho C. Braga, Vinicius M. Alves, Carolina Horta Andrade

**Affiliations:** † Laboratory for Molecular Modeling and Drug Design (LabMol), Faculty of Pharmacy, Universidade Federal de Goiás, Goiânia, GO 74690-900, Brazil; ‡ Center for Excellence in Artificial Intelligence (CEIA), Institute of Informatics, Universidade Federal de Goiás, Goiânia, GO 74690-900, Brazil; § Center for the Research and Advancement in Fragments and Molecular Targets (CRAFT), School of Pharmaceutical Sciences at Ribeirao Preto, University of São Paulo, Ribeirão Preto, SP 05508-220, Brazil; ∥ InsilicAll Inc., São Paulo, SP 04571-010, Brazil; ⊥ University of North Carolina at Chapel Hill, Chapel Hill, North Carolina 27599, United States

## Abstract

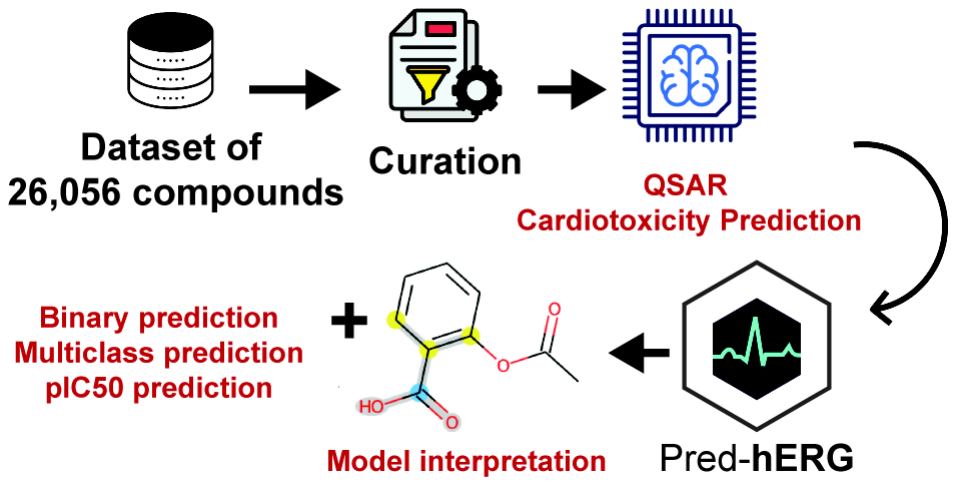

The human Ether-à-go-go-Related Gene (hERG) is a transmembrane protein that regulates cardiac action potential, and its inhibition can induce a potentially deadly cardiac syndrome. *In vitro* tests help identify hERG blockers at early stages; however, the high cost motivates searching for alternative, cost-effective methods. The primary goal of this study was to enhance the Pred-hERG tool for predicting hERG blockage. To achieve this, we developed new QSAR models that incorporated additional data, updated existing classificatory and multiclassificatory models, and introduced new regression models. Notably, we integrated SHAP (SHapley Additive exPlanations) values to offer a visual interpretation of these models. Utilizing the latest data from ChEMBL v30, encompassing over 14,364 compounds with hERG data, our binary and multiclassification models outperformed both the previous iteration of Pred-hERG and all publicly available models. Notably, the new version of our tool introduces a regression model for predicting hERG activity (pIC50). The optimal model demonstrated an *R*^2^ of 0.61 and an RMSE of 0.48, surpassing the only available regression model in the literature. Pred-hERG 5.0 now offers users a swift, reliable, and user-friendly platform for the early assessment of chemically induced cardiotoxicity through hERG blockage. The tool provides versatile outcomes, including (i) classificatory predictions of hERG blockage with prediction reliability, (ii) multiclassificatory predictions of hERG blockage with reliability, (iii) regression predictions with estimated pIC_50_ values, and (iv) probability maps illustrating the contribution of chemical fragments for each prediction. Furthermore, we implemented explainable AI analysis (XAI) to visualize SHAP values, providing insights into the contribution of each feature to binary classification predictions. A consensus prediction calculated based on the predictions of the three developed models is also present to assist the user’s decision-making process. Pred-hERG 5.0 has been designed to be user-friendly, making it accessible to users without computational or programming expertise. The tool is freely available at http://predherg.labmol.com.br.

## Introduction

**Figure 1:**
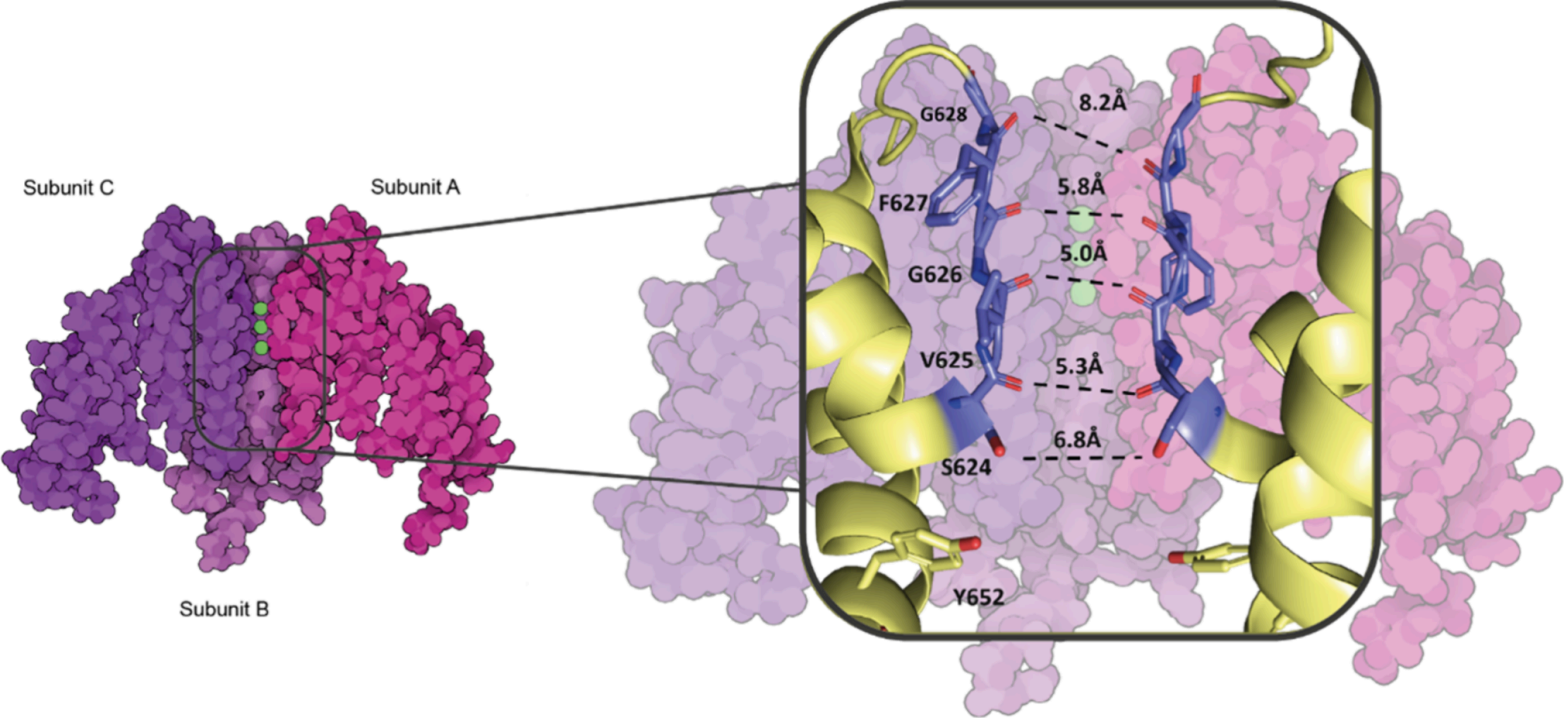
The 3D structure of the hERG channel (PDB ID **7CN0**). Three of its four subunits are displayed, labeled A, B, and C. The amino acids G628, F627, G626, V625, and S624 form the selectivity filter. Although Y652 is not part of the selectivity filter, it plays a role in transporting K+.

The hERG potassium (K+) channel, also known as the human ether-à-go-go-related gene channel (*KCNHA* gene), is a transmembrane potassium channel commonly expressed in the heart, various brain regions, smooth muscle cells, endocrine cells, and a wide range of tumor cell lineages and is responsible for mediating the repolarization current I_kr_, in the cardiac potential, contributing to phase 3 repolarization.^1^ The detailed atomic structure of the channel has recently been elucidated by electron microscopy,^2^ and the channel consists of four identical alpha subunits, which form the transmembrane pore and the K+ selectivity filter, as shown in Figure 1; each hERG subunit consists of six transmembrane alpha-helices, numbered S1–S6.

Several structurally diverse drugs can inhibit the activity of the hERG channel, such as sertindole, terfenadine, cisapride, droperidol and grepafloxacin; these drugs can increase the risk of irregular heartbeat due to the prolongation of the interval between Q and T waves caused by hERG blockage.^3^

hERG-blockade-induced cardiotoxicity is a particular concern due to the increase in postmarket drug withdrawals, which represented 14% of 462 medicinal products withdrawn from the market between 1953 and 2013.^4^ Review shows that cardiac disorders represent 18.8% of 133 drugs started worldwide between 1990 and 2010.^5^ Naturally, this is a broad topic and can be caused by various medications and other related agents, attracting significant attention from scientists and clinicians. Since 2015, regulatory agencies, such as the Food and Drug Administration (FDA), have established requirements to anticipate the discovery of cardiotoxicity in pharmacovigilance.^6,7^

Several recent advancements have led to the development of various *in vitro* assays for evaluating hERG safety. In preclinical settings, telemetry experiments conducted in nonrodents, such as dogs and monkeys, serve as the definitive test for assessing cardiotoxicity, with data generated under physiological conditions and related to the pharmacokinetic profile of the compound.^8^ The conventional patch-clamp electrophysiology in primary cardiac tissue, such as Purkinje fibers, is the preferred *in vitro* method, which allows the evaluation of the electric current passing through hERG channels expressed in cells and provides real-time mechanistic information about the ion channel.^9^ Disadvantages are the cost and time required to carry out such studies. The transgenic zebrafish model has recently been validated as a high-throughput animal model for hERG safety assessment.^10^

Computational methods can help reduce costs and speed up the development and optimization of lead candidates.^23^ Several web servers implementing quantitative structure–activity relationship (QSAR) models using machine learning (ML) and deep learning (DL) algorithms to predict hERG blockage have been published, such as HergSpred,^18^ ToxTree,^19^ CardioTox net,^16^ AMED Cardiotoxicity,^11^ ADMETsar,^12,13^ ADMETlab^14,15^ and CardPred.^17^ Their reported performance, tasks and interpretability are summarized in Table 1.

**Table 1:** Publicly Available Models for hERG Blockage Prediction

Market name	Interpretability	Task	Best Reported Metrics
AMED Cardiotoxicity^11^	No	Regression	*R*^2^ = 0.59
ADMETsar^12,13^	No	Binary and Regression	Binary:AUC = 0.82
ADMETlab^14,15^	No	Binary and Regression	Binary:AUC = 0.94
CardioTox net^16^	No	Binary	ACC = 0.84
CardPred^17^	No	Binary	ACC = 0.9
HergSpred^a^^18^	Yes^a^	Binary	AUC = 0.9
ToxTree^19^	No	Multiclass	ACC = 0.74
Pred-hERG 4.2^20−22^	Yes	Binary and multiclass	BACC = 0.8

a Model interpretability was achieved through a SHAP analysis, which is solely documented in published literature and is not currently accessible within the online tool.

The referenced hERG prediction tools are currently considered state-of-the-art applications that are publicly available. While they have made significant advancements by incorporating robust models and techniques within their platform, such as implementing modern algorithms and interpretable methods, like SHAP (SHapley Additive exPlanations),^24,25^ only the HergSpred^18^ tool employs an interpretation method that is solely documented in published literature and not available within the online tool itself.

An additional issue arises when comparing all available tools: a significant limitation exists in that most of them only offer models for binary prediction, while just two of them, HergSpred^18^ and AMED Cardio,^11^ offer models for multiclass and regression prediction, respectively. This discrepancy highlights a gap in hERG blockage prediction, specifically the lack of robust and interpretable models for binary, multiclass, and regression tasks.

Our group previously developed the Pred-hERG tool,^20−22^ an application that helps researchers identify potential hERG blockers and nonblockers of untested compounds using classificatory (blocker and nonblocker) and multiclassificatory (strong blocker, moderate/weak blocker and nonblocker) QSAR models over the years. The latest version (Pred-hERG 4.2) was released in 2016 and the models were built using data available in ChEMBL v21 with 16,932 chemical records in raw data. The former application was developed using models trained with 8,489 compounds, including 4,437 nonblockers (IC50 ≥ 10 μM), 2,753 moderate/weak blockers (1 μM ≤ activity ≤10 μM), and 1,299 strong blockers (activity ≤1 μM). Both binary (CCR ≈ 0.82) and multiclass models (accuracy ≈0.7) are robust and externally predictive. The tool had a total of 1,147 accesses worldwide over a span of 30 days (from March ninth to April seventh, 2024). The average monthly access rate stands around 580 accesses. Additionally, it has been cited 285 times to date, underscoring its importance within the scientific and pharmaceutical community. While significant advancements have been achieved, the lack of regression models and more robust interpretability analysis restricts a more in-depth analysis of the results, usage and implementation of previous Pred-hERG server.

Considering the recent advancements in automated hERG assays, the subsequent augmentation of publicly accessible hERG data in databases like ChEMBL, the limits of the previous iteration of Pred-hERG and the need for interpretable ML models, updating the preceding iteration of Pred-hERG became imperative. Moreover, incorporating principles from explainable artificial intelligence (XAI)^26−28^ and adding a regression prediction emerges as a viable solution for providing a more in-depth comprehension of Pred-hERG models and a rationalized basis for their predictions.

Therefore, the main goal of this work was to enhance the Pred-hERG tool with new QSAR models, encompassing additional data as well as updating the available binary and multi classificatory models, also integrating new regression models, along with SHAP (SHapley Additive exPlanations)^24,25^ values to estimate the importance of each feature for a given prediction. Overall, our analysis of the mechanistic interpretation with fragment contribution maps and SHAP values provided valuable insights into our machine learning model’s behavior and helped us better understand the factors that influence hERG inhibition activity. This information can guide future research and improve our ability to predict hERG inhibition activity.

## Methods

### Data Collection and Curation

Biochemical hERG data tested in different cell lines were collected from ChEMBL v30^29^ using the ChEMBL ID CHEMBL240, where different bioassay types are reported. Only compounds reporting the half maximal inhibitory concentration (IC_50_) were kept and converted to the pIC_50_ scale (-log IC_50_), in which higher values indicate exponentially greater potency. The data comprised patch-clamp assays performed on the K+ channel (named single protein assay, SP), alongside with cell-based assays conducted on human embryonic kidney 293 cells (HEK293) and Chinese hamster ovary cells (CHO). Chemical structures were retrieved in simplified molecular-input line-entry system (SMILES) format, and according to the workflows outlined by Fourches and colleagues,^30,31^ both chemical and biological data were curated. Briefly, stereoisomers, mixtures and inorganics were removed, salts were cleaned and neutralized, specific chemotypes were normalized, and duplicate records were removed.

For binary and multiclass classification, compounds with IC_50_ values greater than or equal to 10 μM were categorized as inactive. Compounds with a qualifier “>” and IC_50_ values less than 10 μM were excluded. On the other hand, compounds with a qualifier “<” and IC_50_ values less than or equal to 10 μM were deemed active, while those with a qualifier “<” and IC_50_ values greater than 10 μM were eliminated. For the regression models, all compounds with qualifiers such as “>” and “<” were excluded from the data set. This decision was made because the precise value of IC_50_ cannot be determined, rendering this type of data unsuitable for training a regression model.

The duplicate removal process was done in two different ways, contingent upon the specific problem being addressed: (I) binary and multiclass-classification problems had three distinct scenarios, (i) in cases where duplicated records yielded the same outcome, only one record was kept; (ii) when the majority of duplicated records presented the same outcome and one had a different outcome, only one record aligning with the majority is kept; (iii) when duplicated records exhibit varying outcomes, all of them were removed. (II) regression problem, where duplicate records presenting a standard deviation of its pIC_50_ values above 0.2 were removed. The threshold ≤10 μM and >10 μM was used to separate blockers from nonblockers, respectively, while for the multiclass problem, the following was considered: pIC_50_ ≥ 6 for strong blockers, 6 > pIC_50_ ≥ 5 for moderate/weak blockers, and pIC_50_ < 5 for nonblockers. Three distinct data sets were created, each tailored to address a specific problem: binary classification, multiclass classification, and regression. Each of the three data sets underwent a standard split, allocating 80% for training and 20% for testing. This division was executed using the *stratified shuffle split* method in Python v3.8, ensuring consistency with a fixed random state of 42 to facilitate reproducibility. The curated training (80%) and test sets (20%) are provided in the Supporting Information. Furthermore, we conducted a comprehensive analysis of the chemical space to assess the diversity between the training and test sets. The results of this analysis are available in the Supporting Information (Figure S5).

### Correlation and Chemical Space Analysis

The correlation between all three assay activities (HEK, CHO, and Single Protein) was calculated to ensure they had enough statistical correlation before merging all three data sets into one, the method *corr() (which is Pearson by default)* from the library Pandas in Python v3.10 was used. The p-value was also calculated to ensure statistical significance of the results. A *t*-distributed stochastic neighbor embedding (*t*-SNE)^32^ analysis using FCFP4 descriptors with 1024 bits was performed in Python v3.10 to visualize the chemical space of the merged data set. Following the split of the merged data set into training and testing subsets, we conducted a subsequent chemical space analysis. This analysis aimed to evaluate the chemical diversity present within both the training and testing subsets, as well as in comparison to the benchmark data set, and can be seen in the Supporting Information Figure S5. ECFP, FCFP and MACCS descriptors were calculated and a java-based open-source program, SARANEA,^33^ was employed to search for activity cliffs within the data set, where a similarity cutoff of 80% was set.

### QSAR Modeling

The molecular descriptors were computed using open-source chemical descriptors, specifically ECFP-4 and FCFP-4 with 1024 and 2048 bits, respectively, and an atom radius of 2. These calculations were conducted using RDKit and scikit-learn, a Python-based machine learning library. To develop and assess the models, six machine learning algorithms were employed in the scikit-learn library: random forest (RF),^34^ k-nearest neighbors (kNN),^35^ support vector machine (SVM)^36^ and two boosting methods, light gradient-boosting machine (LightGBM)^37^ and extreme gradient boosting (XGBoost).^38^ Model hyperparameters were fine-tuned using Bayesian optimization^39^ (Supplementary Table S1), and a 5-fold cross-validation procedure was conducted on the 80% training set. Following model tuning, the final trained models were evaluated using the remaining 20% of the test set.

The applicability domain (AD) was estimated as defined by Tropsha and Golbraikh.^40^ Also, 20 rounds of Y-randomization were performed to ensure the absence of chance correlations. The Shapley Additive Explanations (SHAP)^24,25^ technique was employed to understand model predictions. The good practices of QSAR modeling defined by the Organization for Economic Cooperation and Development (OECD) were followed.^41^ These practices include (i) defining a specific end point, (ii) using unambiguous algorithms, (ii) defining the applicability domain, (iv) employing appropriate measures to assess robustness and predictivity, and (v), if possible, interpreting the mechanistic aspects of the model.

## Results and Discussion

**Figure 2:**
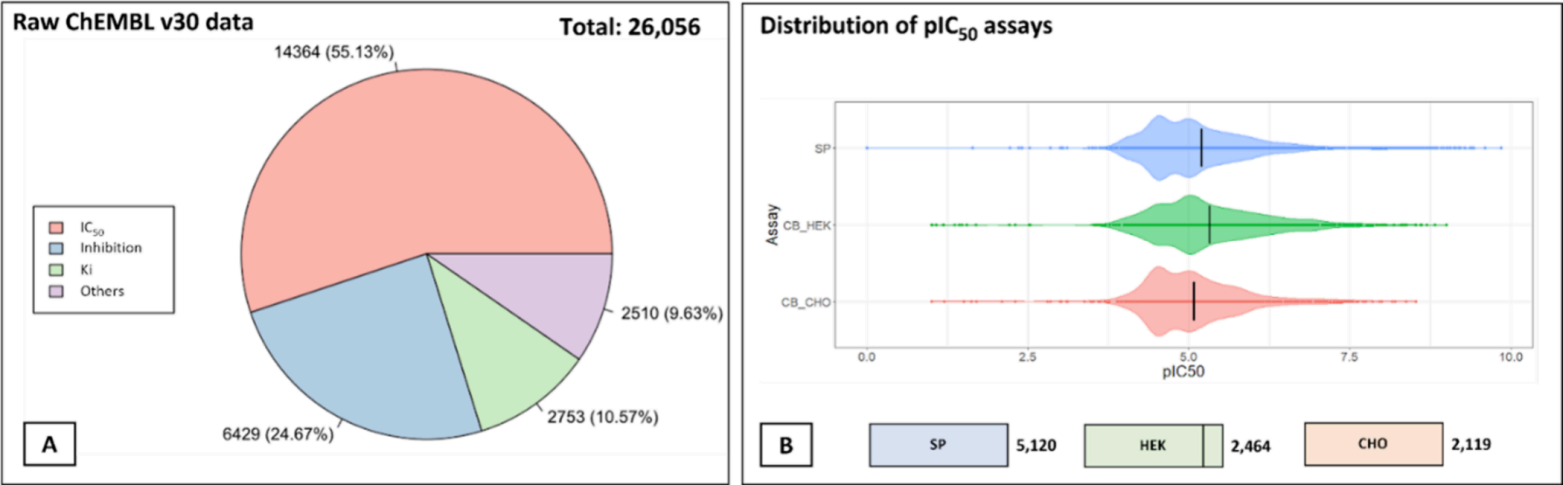
Data distribution of ChEMBL v30 for hERG data. (A) Raw data from ChEMBL v30 database (ID CHEMBL240), where IC_50_ data predominate followed by inhibition (%) data and *K*_i_. (B) The distribution of the pIC_50_ values within the different assay types (SP, HEK CHO), showing most of the compounds in the range of pIC_50_ 4 to 6. SP = Single protein assay. HEK = Human Embryonic Kidney. CHO = Chinese hamster ovary cell.

Data from ChEMBL v30 was collected, and 26,056 compounds were found with hERG activity reported. Of these compounds, 14,364 had IC_50_ values. The second and third most significant values were inhibition (%) and K_i_, respectively, as shown in Figure 2A. The bioactivity distribution of the three assays, cell-based (HEK, CHO) and single protein (SP) is presented in Figure 2B. After the data curation process, only 7,833 compounds remained for the classification tasks and 7,609 for the regression task. (see Supplementary Figure S4 for details on data distribution for the regression data set). The details of the removal process can be found in the Supporting Information (Supporting Table S3).

### Correlation and Chemical Space Analysis

**Figure 3:**
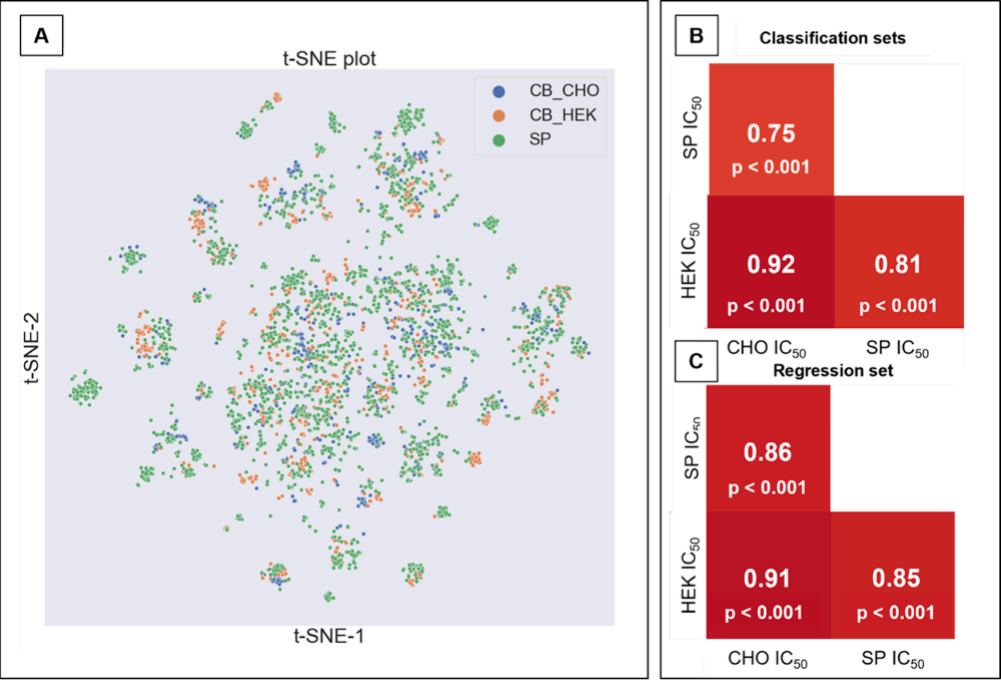
Results of the correlation analysis. (A) Result of the *t-*SNE analysis. The three sets of data, CHO, HEK and SP, were plotted in low dimensionality. It is possible to observe the sparsity of the data; that is, there is no predominant formation of clusters between the data sets, demonstrating greater diversity between the chemical spaces between them. (B and C) Results of the correlation analysis between each data set in relation to the other, as well as the calculated p-value: this analysis is necessary to understand whether there is an experimental correlation between different assays so that all three assays can be merged into a single set for modeling.

Correlation analysis assessed the chemical and activity correlations using Pearson correlation coefficient before merging the HEK, CHO, and SP data sets (Figure 3). The Pearson correlation coefficient was chosen due the data presenting a linear correlation (shown with the calculated p-value) and a normal distribution (which both are required when performing Pearson correlation). We have calculated the p-value using SciPy in Python to test the statistical significance of the correlation. We found the p-value <0.001, indicating that the data exhibits a very high correlation. This result suggests that the null hypothesis (H0) of linear noncorrelation between the comparisons is rejected, given that the p-value is below the chosen significance level. It is shown that the data presents a very high correlation. The obtained correlation results were 0.75 between SP and CHO, 0.81 between SP and HEK, and 0.92 between CHO and HEK, as shown in Figure 3B and Figure 3C. The higher correlation between HEK and CHO can be attributed to both assays being conducted on cell lines, thereby indicating a more substantial relationship in terms of bioactivity. The Tanimoto similarity index was computed by calculating the Tanimoto value for each molecule in a specified data set against every other molecule in another designated data set, namely HEK, CHO, and SP. The resulting mean Tanimoto indices were found to be 0.124, 0.135, and 0.122, respectively. A lower mean Tanimoto index^42^ indicates the dissimilarity between data sets, which is advantageous when creating a QSAR model as it necessitates a chemically diverse data set for accurate modeling.^43^ The *t-*SNE analysis of three assays, SP, HEK, and CHO, was conducted and the resulting map revealed a diverse chemical space with no prominent clusters observed, as seen in Figure 3A.

**Figure 4:**
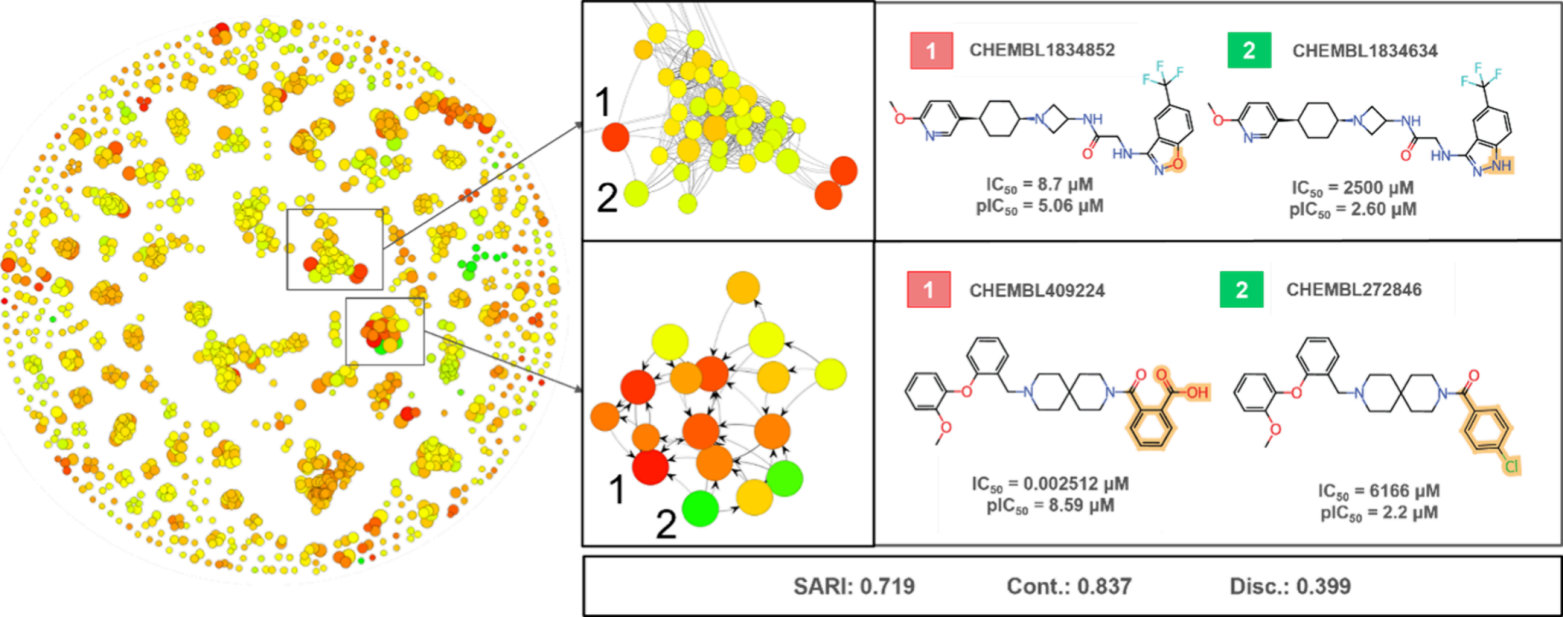
Activity cliffs found in training data using MACCS fingerprints. Activity cliffs CHEMBL183452 and CHEMBL049224 represent blocker compounds (below IC_50_ 10 μM or above pIC_50_ 5), while compounds CHEMBL1834634 and CHEMBL272846 represent nonblocker compounds (IC_50_ above 10 μM or pIC_50_ 5). The colors in the chemical space map represent a scale between nonblockers (green) and blockers (red) and the size of the circles represents the SAR discontinuity of the compound. SARI: Structural Activity Relationship Index; Cont.: continuity score, which is the potency-weighted mean of reciprocal similarity values of a data set. Disc.: Discontinuity score indicates the presence of activity cliffs.

A thorough exploration of the chemical space was performed by employing SARANEA to analyze the data set (Figure 4). This analysis used three different fingerprints: MACCS, ECFP, and FCFP. Among them, MACCS demonstrated the best performance, primarily attributed to its smaller feature set of 166 features. This lower number of features facilitated the program in effectively clustering compounds together. The results revealed a SARI (Structural Activity Relationship Index) value of 0.7 for MACCS, 0.88 for ECFP, and 0.87 for FCFP. SARI is a numerical measure that quantifies the similarity of the molecular structures and biological activities of a pair of compounds. Continuity (cont.) score indicates a continuous, heterogeneous and discontinuous SAR; if its value is high, intermediate or low, respectively. Finally, discontinuity (disc.) indicates the presence of activity cliffs in the data set.^33^

**Figure 5:**
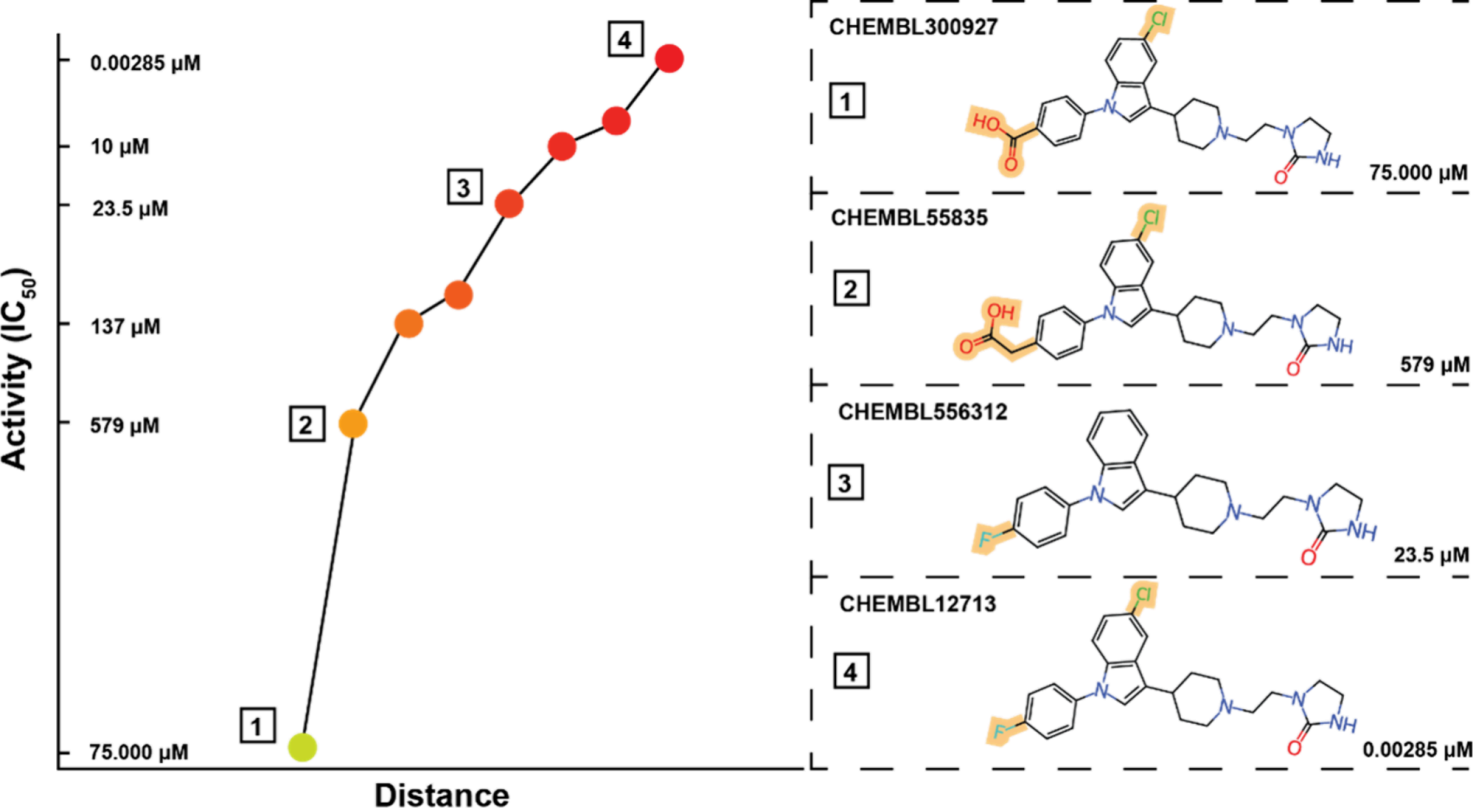
Comparison between small changes in the structure of activity cliffs. It is observed how small changes in the form of these compounds can result in highly different bioactivities; for example, four compounds classified as activity cliffs were represented in this image. Among the molecules, CHEMBL300927 and CHEMBL55835 have organic halides of fluorine and carboxylic acids, as marked in the figure in yellow; however, for CHEMBL55835, the presence of carbon to which the carboxylic acid binds changes the activity from 75,000 μM to 579 μM. For compounds CHEMBL556312 and CHEMBL12713, removing an organic chlorine halide reduced the compound’s activity from 23.5 μM to 0.00285 μM. Graph colors represent activity in green (nonblockers) and red (blockers).

Activity cliffs occur when slight structural differences between a pair of compounds have a large variation in bioactivity, leading to SAR discontinuity. Navigating such discontinuities is difficult for medicinal chemists who are seeking to forecast the impact of structural changes on a molecule’s activity or toxicity, which could result in unexpected and undesirable outcomes during the drug development process. Figure 4 depicts numerous examples of activity cliffs in the context of our SARANEA investigation, underscoring the necessity of understanding and settling these occurrences in drug discovery and predictive toxicology. Notably, the compounds CHEMBL1834852 and CHEMBL1834634 demonstrated how even the slightest alteration, such as replacing a nitrogen atom with an oxygen atom, can substantially shift the bioactivity from IC_50_ = 2500 μM to 8.7 μM. Such sensitivity to minor structural changes poses challenges for creating accurate QSAR models. The subtle nature of these changes makes it difficult for models to effectively differentiate and predict bioactivity accurately, presenting a hurdle in developing robust QSAR models. Some other record examples are depicted in Figure 5, showing the exponential growth in hERG toxicity and its chemical structural changes.

### Model Performance

After careful testing and analysis, the best-performing classification models based on their balanced accuracy (BACC), precision, F1-score, AUC, specificity (Sp), sensitivity (Se), and Matthew’s correlation coefficient (MCC) are shown in Table 2, a regression performance curve and confusion matrices and shown in Supporting Information (Supporting Figure S2 and S3 respectively).

**Table 2:** Results of the Best Models Selected for Activity Prediction against the hERG Channel^a^

Classification Models		BACC	Precision	MCC	AUC	Sp	Se
Binary classification	Training	0.87	0.86	0.52	0.82	0.86	0.85
LightGBM ECFP4 1024	Test	0.86	0.85	0.54	0.81	0.86	0.85
Multiclass classification	Training	0.85	0.83	0.58	0.81	0.85	0.84
RF ECFP4 1024	Test	0.83	0.82	0.56	0.80	0.83	0.82

a BACC: balanced accuracy, AUC: area under the curve; Sp: specificity; Se: sensitivity; MCC: Matthew’s correlation coefficient.

Table 3 shows the best-performing regression model built with ECFP4 1024 descriptors and Support Vector Machine (SVM), based on the coefficient of determination (*R*^2^), mean squared error (MSE), mean absolute error (MAE), and the root-mean-square deviation (RMSE).

**Table 3:** Results of the Best Regression Model Selected for Regression Prediction^a^

Regression model		*R*^2^	MSE	MAE	Experimental MAE	RMSE
SVM-ECFP4 1024	Training	0.63	0.24	0.37	0.28	0.48
	Test	0.61	0.21	0.35		0.44

a *R*^2^: Coefficient of determination, MSE: Mean Squared Error; MAE: Mean Absolute Error; Experimental MAE: MAE calculated between the data set duplicates to achieve experimental error RMSE: Root Mean Squared Error.

The previous versions of Pred-hERG contained only binary and multiclass models,^20−22^ with the latest published in 2015 reporting balanced accuracy of 0.80 and 0.74 for binary and multiclass, respectively. The new models reported herein showed a slight increase of ≅ 0.07 for binary classification and a more significant increase of ≅ 0.11 for multiclass classification. The new models were trained on a significantly larger data set comprising 7,833 records for classification and 7,609 for regression. This represents a significant increase compared to the 5,984 chemical records utilized in the previous version.

Additionally, the training data set underwent more thorough curation using Fourches and colleagues^30,31^ protocols, particularly in addressing activity cliffs which can significantly hinder model performance and generalization, as discussed below, which significantly improved model performance in the three tasks. We also calculated the MAE between duplicates of the regression data set before removing them to obtain the experimental error (Table 3). When comparing the experimental MAE of 0.28 to the predicted RMSE of 0.44, we can see the MAE is significantly lower than the RMSE, which indicates the predictions align well with experimental values. Upon comparing the models developed in this project with those previously reported, noticeable improvements in predictive capabilities can be observed.

Pred-hERG 5.0 is currently the sole tool that integrates three learning tasks (binary, regression, and multiclass) and features a user-friendly interface alongside interpretable models. With the improvements presented in this new version, Pred-hERG endeavors to address the deficiency in hERG blockage prediction. This deficiency pertains specifically to the scarcity of robust and interpretable models suitable for binary, multiclass, and regression tasks.

To facilitate user decision-making regarding the prediction of a molecule as either a blocker or nonblocker, we conducted an analysis of agreement among three models using Cohen’s kappa (Supplementary Figure S3). Given the models exhibited an acceptable agreement score, our tool will furnish a weighted ensemble outcome. This outcome is derived by assigning varying weights to the predictions of each model: 0.6 for binary, and 0.2 for regression and multiclass predictions when they concur, and 0.1 when they do not agree, as delineated in eq 1. The agreement of the regression and multiclass model is based on converting the outcome of the regression model into multiclass labels. This transformation adheres to the following rule: compounds are categorized as strong blockers if their pIC50 values are greater than or equal to 6, as moderate if their pIC50 values fall between 6 and 5, as weak if their pIC50 values range from 5 to 4.5, and as nonblockers if their pIC50 values are less than 4.5.
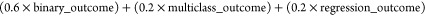
1The output value of this function will be used to determine whether a molecule is a blocker or not. Several thresholds were tested, and the best performing threshold with balanced accuracy of 0.82 is 2, in this case, if the final outcome ≥2, then the output will be blocker, if the final outcome <2, then the output will be nonblocker. Confusion matrix of the weighted consensus outcome can be seen in Figure S2 of the Supporting Information. This will help users make better informed decisions, alongside with the individual prediction of each binary, multiclass and regression model, as well as the interpretation available with SHAP and Probability maps.

**Figure 6:**
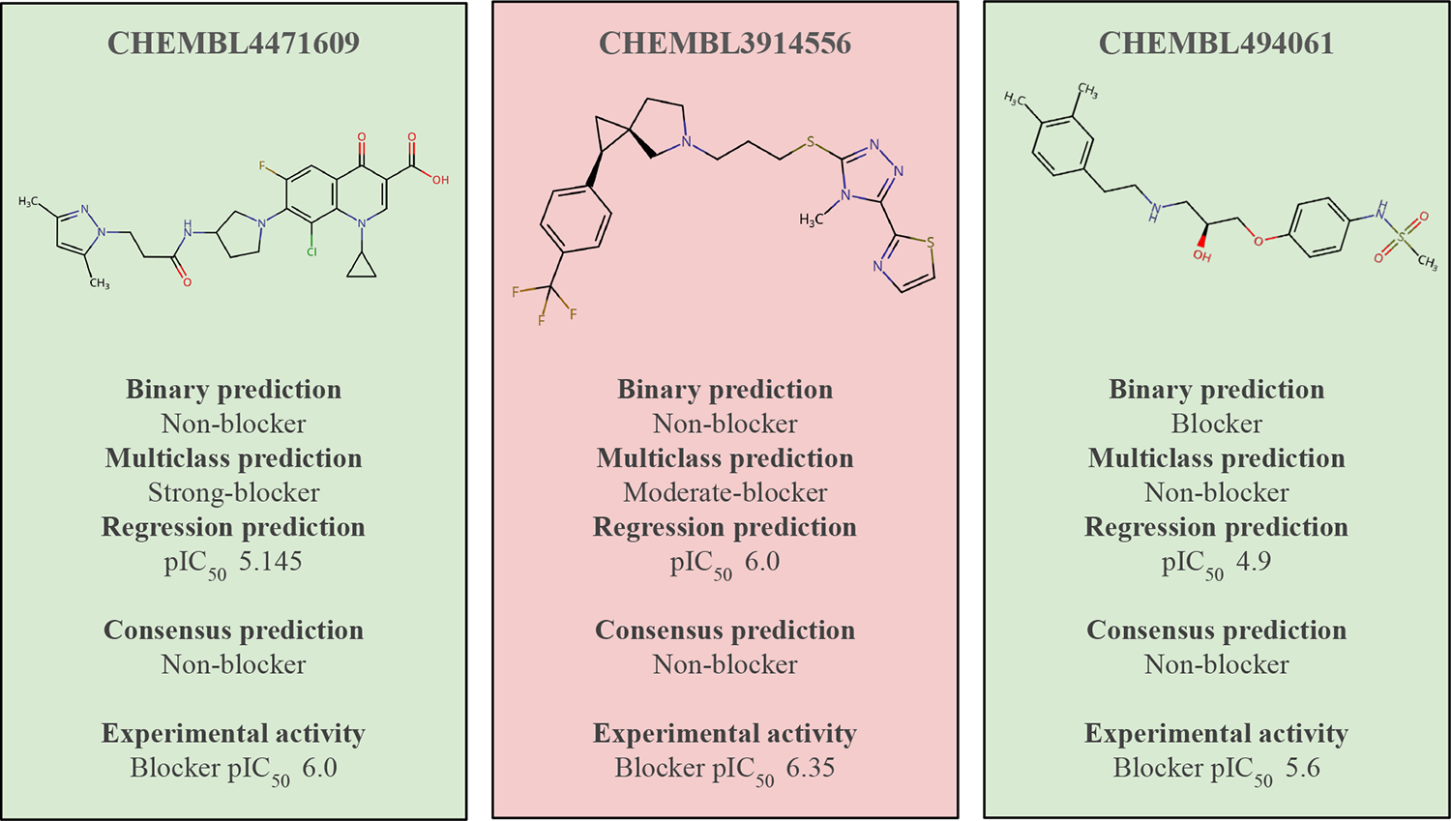
Weighted consensus outcome mispredictions. Three examples of mispredictions caused by the weighted consensus are shown above. CHEMBL4471609, CHEMBL3914556 and CHEMBL494061.

We conducted an in-depth assessment of mispredictions by analyzing the weighted consensus outcome. Our findings, detailed in Figure 6, indicate that the majority of mispredictions occur when the consensus outcome diverges from the collective decision of the three models, as depicted in Figure 6. This discrepancy is primarily attributed to the weighted nature of the output, where the binary classification model, given its higher weight, often steers the final prediction toward “blocker” rather than “nonblocker”, as evidenced by instances like CHEMBL4471609 (Figure 6).

Moreover, in certain cases, both the multiclass and regression models incorrectly predict “blocker” instead of “nonblocker”, as seen with CHEMBL3914556 and CHEMBL494061 (Figure 6). Addressing this issue may entail adopting a more effective consensus method. Importantly, such a modification does not necessitate retraining the models, prompting our exploration of alternative methods and their potential applications. Furthermore, our future research will encompass investigating diverse machine learning approaches, leveraging insights from this study, including the impact of specific features and activity cliffs within the data set. Our overarching goal is to develop refined and robust models that significantly enhance hERG blockage prediction.

**Figure 7:**
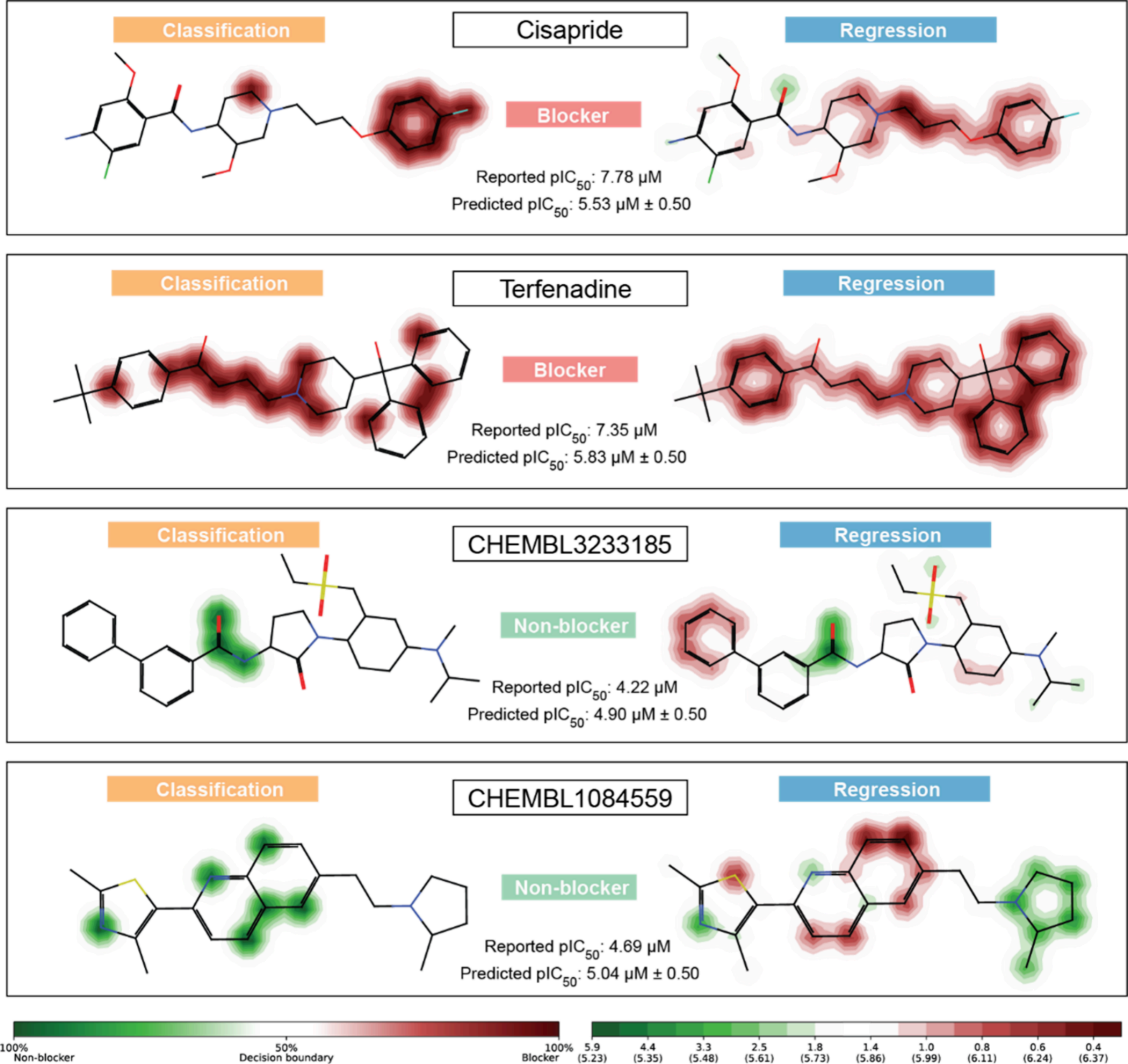
Predicted probability maps generated by classification and regression models. Two known blockers (Cisapride and Terfenadine) and two experimentally validated nonblockers (CHEMBL3233185 and CHEMBL1084559). The fragments in red represent structures that influence the model to predict the molecule as cardiotoxic; in this case, the more intense the red, the more positive contribution to the prediction that fragment will have the same goes for fragments in green, which represent negative contributions to the model’s prediction.

Pred-hERG 5.0 generates probability prediction maps that illustrate the contribution of individual fingerprint atoms to the predictive probability of machine learning models. These maps facilitate the comprehension of how a specific fragment can impact the compound’s activity, either positively or negatively. This valuable insight aids in developing novel compounds with reduced hERG toxicity by guiding the design process. Figure 7 shows the probability prediction maps with different cardiotoxic profiles for known blockers and nonblockers and their predictions by Pred-hERG 5.0, where the fragments contoured in red represent fragments that influence the model to predict the molecule as cardiotoxic and fragments contoured in green represent negative contributions to the model’s prediction.

Several tools are freely available to predict hERG blockage, mainly from academic groups. However, as shown in Supporting Information (Supporting Table S2), the majority of the published QSAR models do not adhere to the standard validation protocols and statistical criteria outlined in the best practices of QSAR modeling nor are they compliant with the OECD guidance on QSAR model development and validation.^41^ For instance, (i) most models lack proof of passing the Y-randomization test,^11,16,19,44−51^ (ii) no evidence is provided for applicability domain (AD) estimation,^11,16,19,44,48−51^ (iii) model predictivity is unacceptable,^11,16,45,49^ and (iv) implementing the best practices to curate the data sets are not described, as proposed by Fourches and collaborators.^30,31^ As a result, despite the availability of many QSAR models for hERG blockage in the literature, only a few models could be used in practice. Furthermore, most models and associated data sets used to develop them are unavailable to the scientific community.

A critical examination of recent models reveals that most exhibit the same failures as those reported in previous studies.^21,52^ These significant drawbacks jeopardize the practical application of these models for the reliable assessment of drug-induced QT syndrome. A few enhancements are worth mentioning. Some models used more sophisticated algorithms to predict the inhibition of hERG K^+^ channels, such as implementing deep neural networks (DNN), convolution neural networks, and optimized machine learning algorithms.^16,19,44,48,50,53,54^ Also, diverse molecular descriptors were applied to obtain a better chemical representation from molecules and add protein–ligand information to teach the algorithm to differentiate minor differences between the molecules.^16,45,46,49,50,54,55^

The two papers published by Siramshetty and colleagues^46,47^ clearly shows the application of DNNs and latent descriptors to improve model performance. The results show that machine learning algorithms outperform DNN, but using latent descriptors appears to better distinguish between blockers and nonblockers of hERG K+ channels. Wang and colleagues^51^ proposed a promising DNN alternative to improve performance by employing convolution-capsule networks and restricted Boltzmann capsule networks. These approaches showed increased prediction while outperforming machine learning developed in other scientific papers. Even though these models perform well, the authors did not report Y-randomization results or the applicability domain estimation in their papers. Only Wacker and Noskov^54^ presented these preconized methodologies, which unfortunately did not perform well.

### Model Interpretation with XAI Analysis

**Figure 8:**
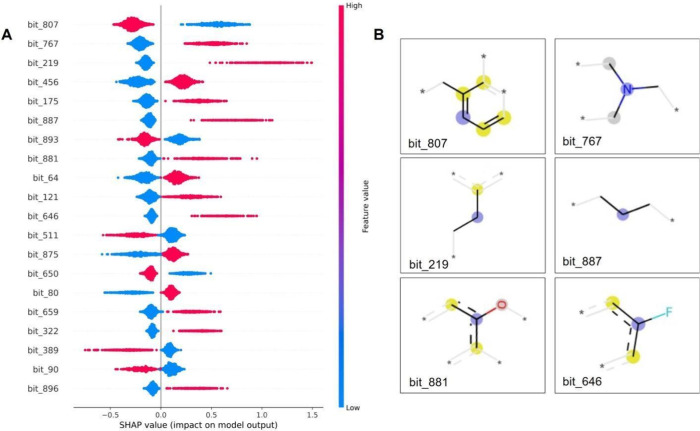
Global SHAP interpretation of the binary model LightGBM ECFP4 1024. (A) SHAP values, with the *x*-axis indicating their impact on the model output and the *y*-axis representing feature values. In the plot, red denotes a positive impact, while blue signifies a negative impact on the model prediction. (B) Most frequently occurring fragments: 807, 787, 219, 887, 881, and 646, represented as fragments on the right (Figure 8B). Blue contour atoms: represent the central atom in the environment; yellow: aromatic atoms; gray: aliphatic ring atoms.

We calculated the SHAP values to interpret our classification machine learning model’s feature importance for the binary model. Due to the high requirement of computer power, the online tool Pred-hERG 5.0 only computes SHAP interpretation for the binary model. SHAP values provide a measure of the contribution of each feature to the prediction for a given instance. One can identify the features with the most impact on model output by analyzing the SHAP values. Figure 8 shows an example, where bits 807, 767, and 219 were among the most relevant features for binary classification. These features had high average absolute SHAP values across all instances, indicating that they strongly influenced the model’s predictions. The presence of bits 807 and 650 showed a higher impact on the model prediction of nonblockers, while bits 767, 219, 175, 887, 881, 121, and 646 showed a higher impact on the model prediction of blockers. Since a circular fingerprint of radius 4 was used, many features represented lack any real chemical significance on their own, but a more funneled analysis can still be done with local SHAP.

**Figure 9:**
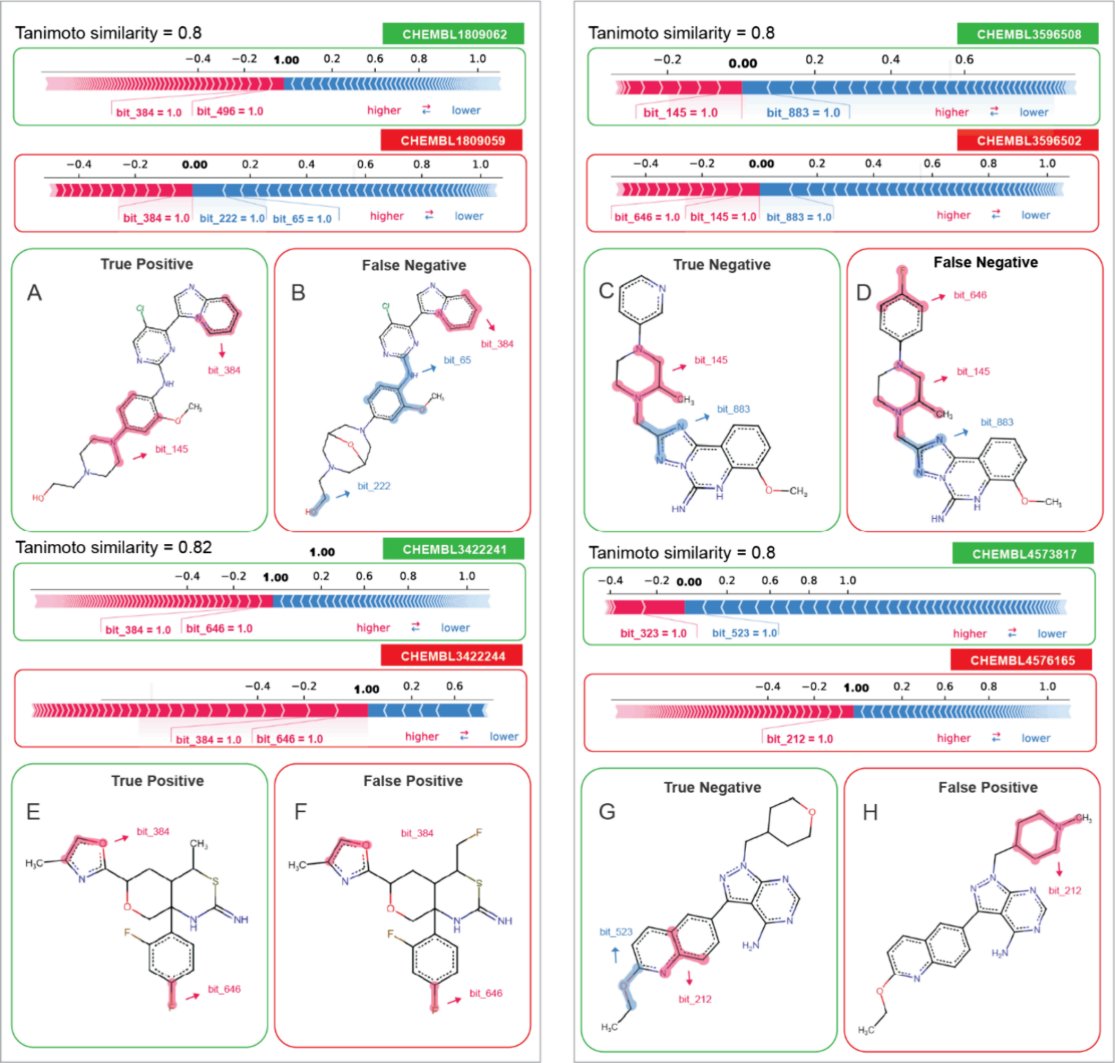
Local SHAP interpretation of the binary model LightGBM ECFP4 1024 in highly similar chemical pairs. The figure illustrates eight examples of activity cliffs, i.e., highly similar chemical pairs with opposed outcome. Compounds highlighted with green squares were accurately predicted, while those marked with red squares denote incorrect predictions. The SHAP fragments in red indicate a negative impact on the model’s prediction, and fragments in blue represent a positive influence on the model’s prediction.

A more in-depth examination of the binary model’s predictions through local SHAP analysis highlighted the significant impact of activity cliffs in contributing to prediction errors. As depicted in Figure 9 we scrutinized pairs of compounds with a high Tanimoto similarity score but opposing outcomes (blocker and nonblocker). As an example, the compounds depicted in Figure 9E-9F exhibit a difference solely in the substitution of a methyl group with fluoroethane, leading to a nonblocker outcome in the experimental results for the compound CHEMBL3422244. Notably, bits 384 and 646, the most relevant bits for these molecules, fail to capture the methyl-to-fluoroethane modification. This deficiency suggests that the model may struggle to adequately interpret this specific modification, thereby contributing to a prediction error. Another example involves the substitution of an oxane ring by a 1-methylpiperidine (Figure 9G-9H), resulting in a nonblocker. Interestingly the SHAP analysis showed no correlation between the bits present in the molecule pair in Figure 9G-9H. This discrepancy could be from the nuanced nature of local analysis, where different bits hold varying levels of significance even for closely related molecules. Once again, the model struggles to interpret the subtle modification between these molecules, leading to a prediction error. A similar challenge is observed between the pair of molecules depicted in Figure 9A-9B, where no shared bits are identified, except for bit 384, which contributes to the model’s incorrect prediction for CHEMBL1809059. Lastly, in Figure 9C-9D, most bits are shared between the pair of molecules, except for bit 646 present in molecule CHEMBL3596502. Both bits 145 and 646 in this molecule contribute to an inaccurate prediction by the model.

The examples depicted in Figure 9 underscore the challenges the model faces in capturing the nuanced impacts of subtle structural variations on molecular activity. This analysis facilitates a more comprehensive understanding of the factors influencing a molecule’s prediction and subsequently aids in interpreting the model output. Given the prevalence of activity cliffs within the hERG training data set, as shown in Figures 4 and 5, modeling the hERG end points becomes particularly challenging. Alternative approaches to address this issue will be investigated in future endeavors, utilizing large-language models, attention mechanisms and feature engineering. Additionally, future studies may explore the utilization of the 3D structure of the hERG channel recently solved^2^ to probe the correlation between the presence of specific fragments and hERG blockage.

### Benchmark

We evaluated the performance of Pred-hERG 5.0 in predicting hERG blockage for an external data set of 820 compounds collected from ChEMBL v33 (ID CHEMBL240). This data set does not contain data used during the training or validation of the models. Table 4 and Table 5 shows the performance of Pred-hERG 5.0 compared with seven state-of-the-art available QSAR models for the binary classification and regression tasks (ADMETlab,^14,15^ CardPred,^17^ ADMETsar,^12,13^ HergSPred,^18^ AMED Cardio,^11^ Cardiotox net,^16^ and DeepHIT^44^).

**Table 4:** Statistical Characteristics of the Binary Classification Models Predictions on an External Set of 840 Compounds (CHEMBL240)

Model	BACC	SE	SP	MCC	F1	AUC
ADMETlab^14,15^	0.8	0.75	0.84	0.62	0.6	0.68
CardPred^17^	0.72	0.7	0.74	0.45	0.68	0.56
ADMETsar^12,13^	0.76	0.68	0.84	0.49	0.6	0.67
AMED cardio^11^	0.8	0.78	0.81	0.56	0.72	0.78
Cardiotox net^16^	0.77	0.8	0.75	0.48	0.67	0.71
DeepHIT^44^	0.81	0.78	0.85	0.59	0.71	0.73
**Pred-hERG 5.0**	**0.82**	**0.82**	**0.83**	**0.65**	**0.74**	**0.82**

**Table 5:** Statistical Characteristics of the Regression Model on an External Set of 840 Compounds (CHEMBL240)

Model	*R*^2^	MSE	RMSE	MAE
AMED Cardio (11)	0.56	0.32	0.54	0.48
**Pred-hERG 5.0**	**0.66**	**0.21**	**0.45**	**0.35**

As shown in Table 4, Pred-hERG 5.0 demonstrated higher accuracy compared to all five available models in predicting hERG blockage within the binary models. Unfortunately, we were not able to compare our multiclass model with the HergSpred^18^ server, because it was unavailable when we performed this analysis. Pred-hERG also underwent evaluation in a regression task (Table 5) and it outperformed the regression model available AMED Cardio.^11^

## Conclusions

In conclusion, this work successfully incorporated new classificatory and multiclassificatory QSAR models developed with almost the double of compounds in the curated data set in comparison to previous versions of Pred-hERG, being the first tool to integrate all three tasks (classification, multiclass and regression) into a weighted consensus prediction as well as easy-to-use interpretation of the results using SHAP and probability maps. The inclusion of regression models and the implementation of SHAP values for interpretability, marks a significant advancement in the Pred-hERG platform. Utilizing the latest data from ChEMBL v30, encompassing over 14,364 compounds, the newly developed classification models outperform all previous public models, demonstrating robustness with BACC, AUC, and MCC values of 0.86, 0.81, and 0.54, respectively. The multiclassification model, utilizing FCFP2 descriptors and the Random Forest algorithm, achieves notable BACC, AUC, and MCC values of 0.83, 0.81, and 0.64, respectively. Furthermore, the introduction of a regression model for predicting hERG activity (pIC_50_) sets a new standard, surpassing the existing literature model with an *R*^2^ of 0.61 and an RMSE of 0.48. Therefore, Pred-hERG 5.0 emerges as a fast, reliable, open-source and user-friendly tool for early assessment of chemically induced cardiotoxicity via hERG blockage. Users benefit from classificatory and multiclassificatory predictions, regression predictions with pIC_50_ values, and probability maps illustrating chemical fragment contributions. Moreover, the tool’s model interpretation is enhanced through the integration of explainable AI analysis (XAI) using SHAP values, offering insights into feature contributions for binary classification predictions. Importantly, Pred-hERG 5.0 is readily accessible to users without the need for computational or programming expertise, and it can be freely accessed at http://predherg.labmol.com.br. Additionally, all scripts utilized in this study are accessible at https://github.com/LabMolUFG/Pred_hERG, and we welcome future feedback to enhance the development of the tool.

## Supporting Information Available

Supporting Information 1: Supporting tables with hyperparameters used during model training (Table S1), published hERG models available in the literature from 2016 to 2023 (Table S2) and details of the data removed during the curation process (Table S3). Figure S1 with Regression *R*^2^ plots for the test data set. Figure S2 with Confusion matrices for binary and regression as well as the weighted consensus outcome. Figure S3 with Cohen’s kappa concordance test between all three models. Figure S4 with Histogram of data distribution within regression model training data set and Figure S5 with Chemical space analysis using t-SNE between test, train and benchmark data set (PDF)
Supporting Information 2: all databases used in the modeling (binary, multiclass and regression, as well as for the benchmark) (ZIP)
