# Supplementary material for: Enhancing hERG Risk Assessment with Interpretable Classificatory and Regression Models
Source: Chem Res Toxicol. 2024 May 23;37(6):910–22. doi: 10.1021/acs.chemrestox.3c00400 (PMC11187631; doi:10.1021/acs.chemrestox.3c00400)
Supplement: Supplementary file 1 — tx3c00400_si_001.pdf [file tx3c00400_si_001.pdf]

Enhancing hERG Risk Assessment with Interpretable Classificatory and Regression Models

Igor H. Sanches,[1,2,3] Rodolpho C. Braga,[4] Vinicius M. Alves[5] and Carolina Horta Andrade[1,2,3]*

[1]Laboratory for Molecular Modeling and Drug Design (LabMol), Faculty of Pharmacy, Universidade Federal de Goiás, Goiânia, GO, Brazil.

[2]Center for Excellence in Artificial Intelligence (CEIA), Institute of Informatics, Universidade Federal de Goiás, Goiânia, GO, Brazil.

[3]Center for the Research and Advancement in Fragments and Molecular Targets (CRAFT), School of Pharmaceutical Sciences at Ribeirao Preto, University of São Paulo, Ribeirão Preto, SP, Brazil.

[4]InsilicAll Inc., São Paulo, SP, Brazil.

[5]University of North Carolina at Chapel Hill, Chapel Hill, NC, USA.

Corresponding Author * Address for correspondence: Laboratory for Molecular Modeling and Design, Faculty of Pharmacy, Federal University of Goiás, Goiânia, GO, 74605-170, Brazil; Telephone: +55 62 3209-6451; FAX: +55 62 3209-6037. E-mail: carolina@ufg.br.

Table of contents

Hyperparameters tuned during model training S3

Published hERG models from 2016 to 2023. S4

Data removed during the curation process. S8

Regression R^2 plots for the regression model S9

Confusion matrices for binary and regression models as well as the weighted consensus outcome S10

Cohen's kappa concordance test between all three models S11

Histogram of data distribution within regression model training and test dataset S12

Chemical space analysis using tSNE between test, train and benchmark dataset S13

Table S1. Hyperparameters tuned during model training.

Model	Hyperparameters tuned
Xgboost	learning_rate, max_depth, subsample, colsample_bytree, reg_alpha, n_estimators
LGBM	reg_sqrt, learning_rate, n_estimators, num_leaves, max_depth, subsample, subsample_freq, colsample_bytree, reg_lambda, reg_alpha
SVM	C, gamma and kernel
kNN	n_neighbors, weights, metric
Adaboost	learning_rate, n_estimators
RF	max_features, n_estimators, max_depth, min_samples_leaf, min_samples_split

Table S2. Published hERG models from 2016 to 2023.

Nº compounds (training)	Nº compounds (external)	Dataset (training/external)	Curation	Activity	Activity threshold	Performance (External test set unless otherwise specified)		AD	Y-rand	Features	Availability	Year	References
421	External test = 309<br>In silico test = 7361	Misc	No	Ki	Inactive 5-54 µM<br>Moderate 50nM-5µM<br>Potent ≤ 50nM	QSAR-3D	training set = Q^2 0.80<br>external set = r^2 0.86<br>In silico test = ROC 0.96	No	No	Physicochemical properties	No	2017	1
700	283	ChEMBL v23	No	pIC50	> 5 active	Lasso<br>SVM<br>RF<br>XGBM	MAE 0.80<br>R^2 0.61<br>RMSE 0.98	Yes	Yes	Pharmacophore features (FS2)	No	2017	2
8380	499	ChEMBL v22/ Misc	Yes	pIC50	≥ 6 strong blocker<br>6 > pIC50 ≥ 5 moderate blocker<br>5 > pIC50 ≥ 4.5 weak blocker	DNN<br>Kernelized-SVM<br>RF	Q4 0.75<br>Q2 0.94<br>SE 0.99<br>SP 0.75<br>MCC 0.80<br>CCR 0.87	No	No	PaDEL-Descriptor v2.21	Yes	2021	3

					Pic50 < 4.5 non-blocker								
14,831	700	ChEMBL v23 / Misc	No	IC50/Ki	≤ 1μM - blocker<br>> 10μM - non-blocker	RF	AUC 0.97<br>ACC 0.93<br>SE 0.87<br>SP 0.97<br>MCC 0.85<br>BA 0.92	Yes	No	PubChem and Morgan fingerprints	No	2018	4
12620	924	Misc	Yes	IC50	> 10μM nonblocker	DNN	MCC 0.599<br>NVP 0.688<br>ACC 0.810<br>PPV 0.893<br>SPE 0.786<br>SEN 0.833	No	No	Node feature and<br>adjacency vector	Yes		5
8337	100 (random f/ training set)	ChEMBL v25	No	IC50	≤ 1μM blocker	Lasso SVM	ACC 0.79<br>NPV 0.80<br>AUC 0.86	No	No	Interaction Fingerprint (i. e., MOLPRINT2D)			6
6561	4630	Misc / randomly from train	Yes	IC50	Blocker if<br>≤10μM or	SVR	R 0.72<br>R^2 0.28<br>RMSE 0.87	No	No	Morgan, Mordred, and RDKit fingerprint	Yes	2021	7

		set			≥50% inhibition at 10μM	RF	R 0.73<br>R^2 0.53<br>RMSE 0.67			s			
						DNN	R 0.69<br>R^2 0.48<br>RMSE 0.68						
233	27	Misc	No	pIC50	NA	PLS regression	R^3 0.72<br>RMSD 0.7	No	No	Protein Ligand Interaction fingerprint	No	2021	8
9215	1146	ChEMBL v29 / Misc	No	pIC50	NA	SVR	PCC 0.765<br>RMSE 0.58<br>Spearman's ρ 0.757	Yes	No	Morgan,<br>MACCS, and<br>Estate fingerprints	No	2021	9
2644	60	Misc	No	IC50	< 10 μM blocker<br>>30 μM nonblocker	Capsule Networks	SE 0.89<br>SP 0.89<br>MCC 0.774<br>SD 0.0109<br>Q 0.89	No	No	MACCS fingerprints	No	2020	10
14440	44	Misc	Yes	IC50	< 10 μM blocker<br>≥10 μM<br>nonblocker	DNN	ACC 0.773<br>MCC 0.476<br>SE 0.833<br>NPV 0.643	No	No	Mordred, Morgan, and Pubchem fingerprints	Yes	2020	11

1428	4095	Misc	Yes	IC50	IC50 ≤ 40μM blocker	Pharmacophore Modelling	TP:119/177 TN:68/177 FN:24/32 FP:6/32 MCC=0.72	Yes	No	Grid Independent Molecular Descriptor (GRIND)	No	2018	12
8154	1016	Misc	No	IC50	between 1 and 10μM are removed	Consensus (RF, XGBoost, DNN, LSTM)	BACC 0.80 SEN 0.74 SP 0.86	No	No	RDKit and Morgan fingerprints	No	2020	13

Table S3. Data removed during the curation process.

Assay	*CHO*			*HEK*			hERG K+ channel		
Number of compounds/type of model	*Multiclass*	*Binary*	*Regression*	*Multiclass*	*Binary*	*Regression*	*Multiclass*	*Binary*	*Regression*
Initial	*5.120*	*5.120*	*5.120*	*2.464*	*2.464*	*2.464*	*2.119*	*2.119*	*2.119*
Removed due to lack of activity values	*276*	*276*	*276*	*164*	*164*	*164*	*242*	*242*	*242*
Organometallics	*0*	*0*	*0*	*0*	*0*	*0*	*0*	*0*	*0*
Mixtures	*0*	*0*	*0*	*0*	*0*	*0*	*0*	*0*	*0*
Removed during the relationship preparation process	*99*	*99*	*224*	*18*	*18*	*130*	*173*	*173*	*229*
Concordant duplicates	*406*	*406*	*398*	*224*	*224*	*203*	*118*	*118*	*98*
Discordant duplicates (removed)	*42*	*42*	*38*	*21*	*21*	*18*	*12*	*12*	*9*
Total duplicates	*448*	*448*	*436*	*245*	*245*	*221*	*130*	*130*	*107*
Final value	***4.255***	***4.255***	***4.146***	***2.016***	***2.016***	***1.931***	***1.562***	***1.562***	***1.532***

Figure S1 – Regression R^2 plots for the train and test sets

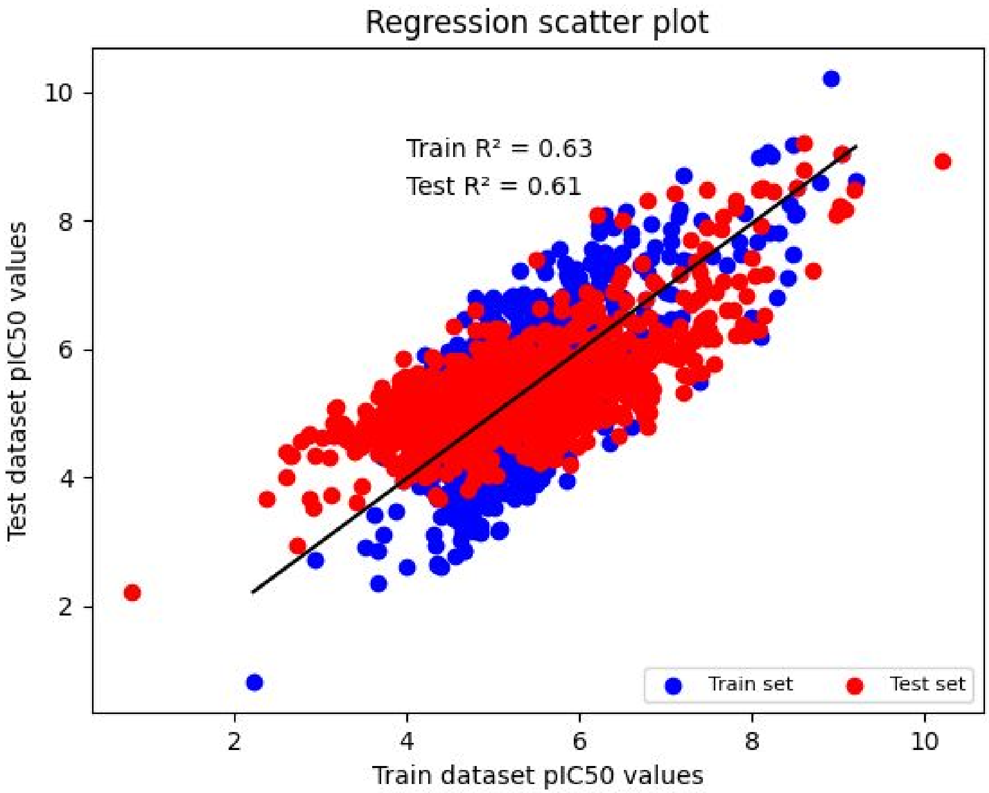

Figure S2 – Confusion matrices for binary and multi-class models as well as the weighted consensus outcome.

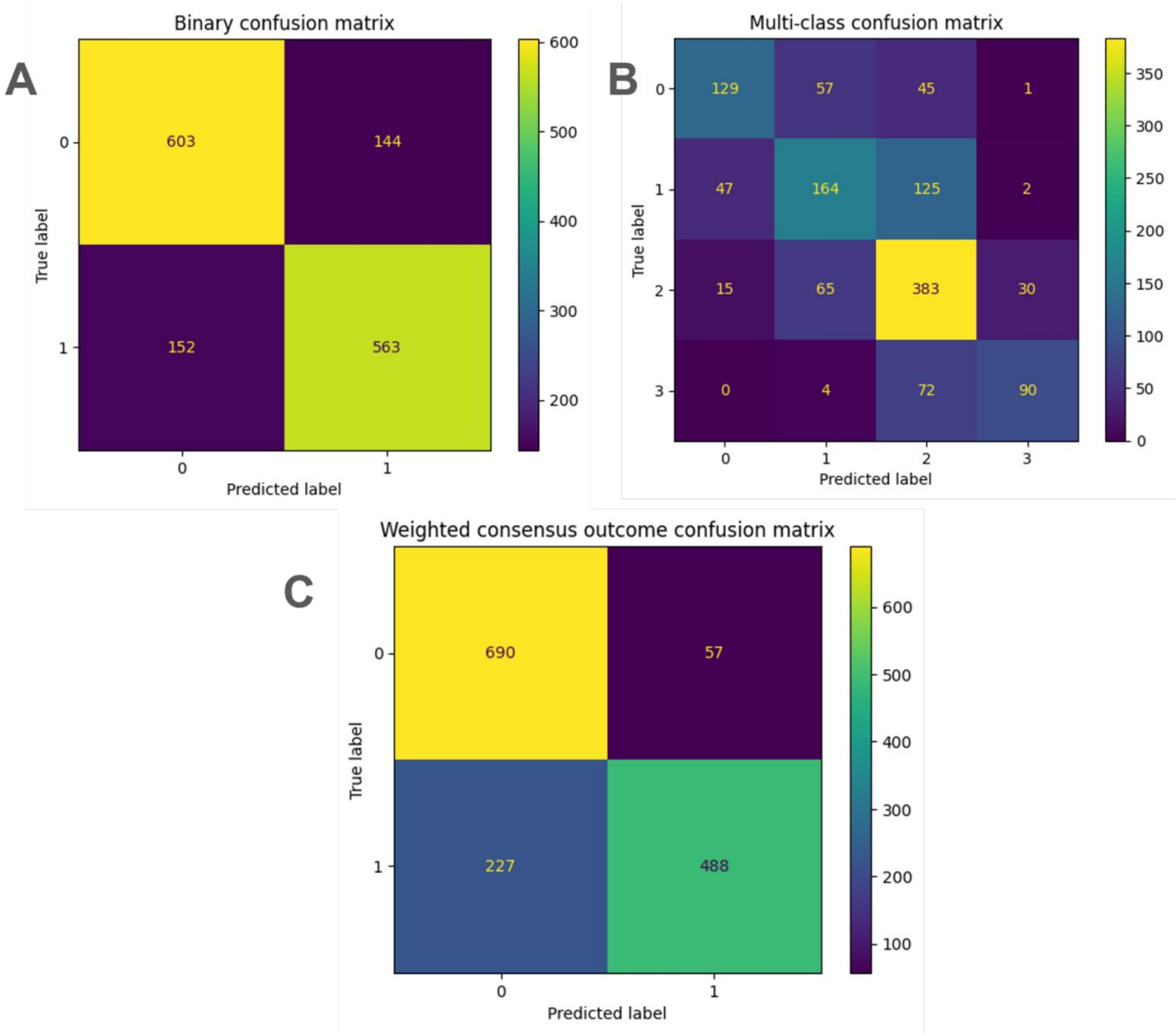

A
Binary confusion matrix
603
144
152
563
True label
Predicted label
B
Multi-class confusion matrix
129
57
45
1
47
164
125
2
15
65
383
30
0
4
72
90
True label
Predicted label
C
Weighted consensus outcome confusion matrix
690
57
227
488
True label
Predicted label

Figure S3 – Cohen's kappa concordance test between all three models.

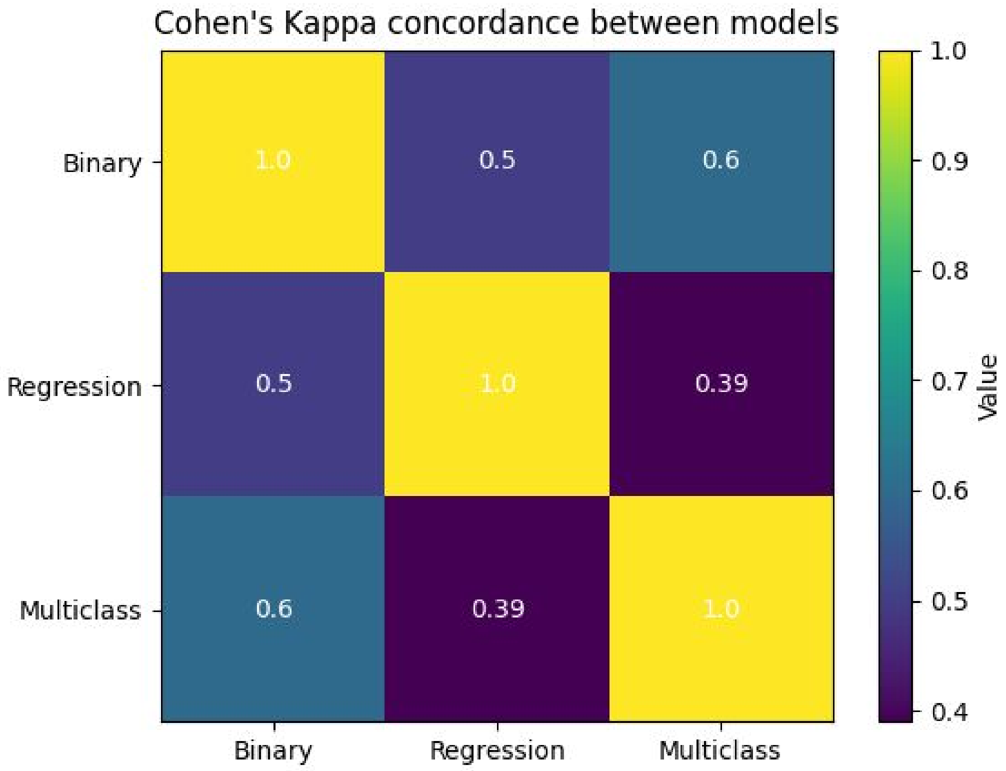

Figure S4 – Histogram of data distribution within regression model training dataset

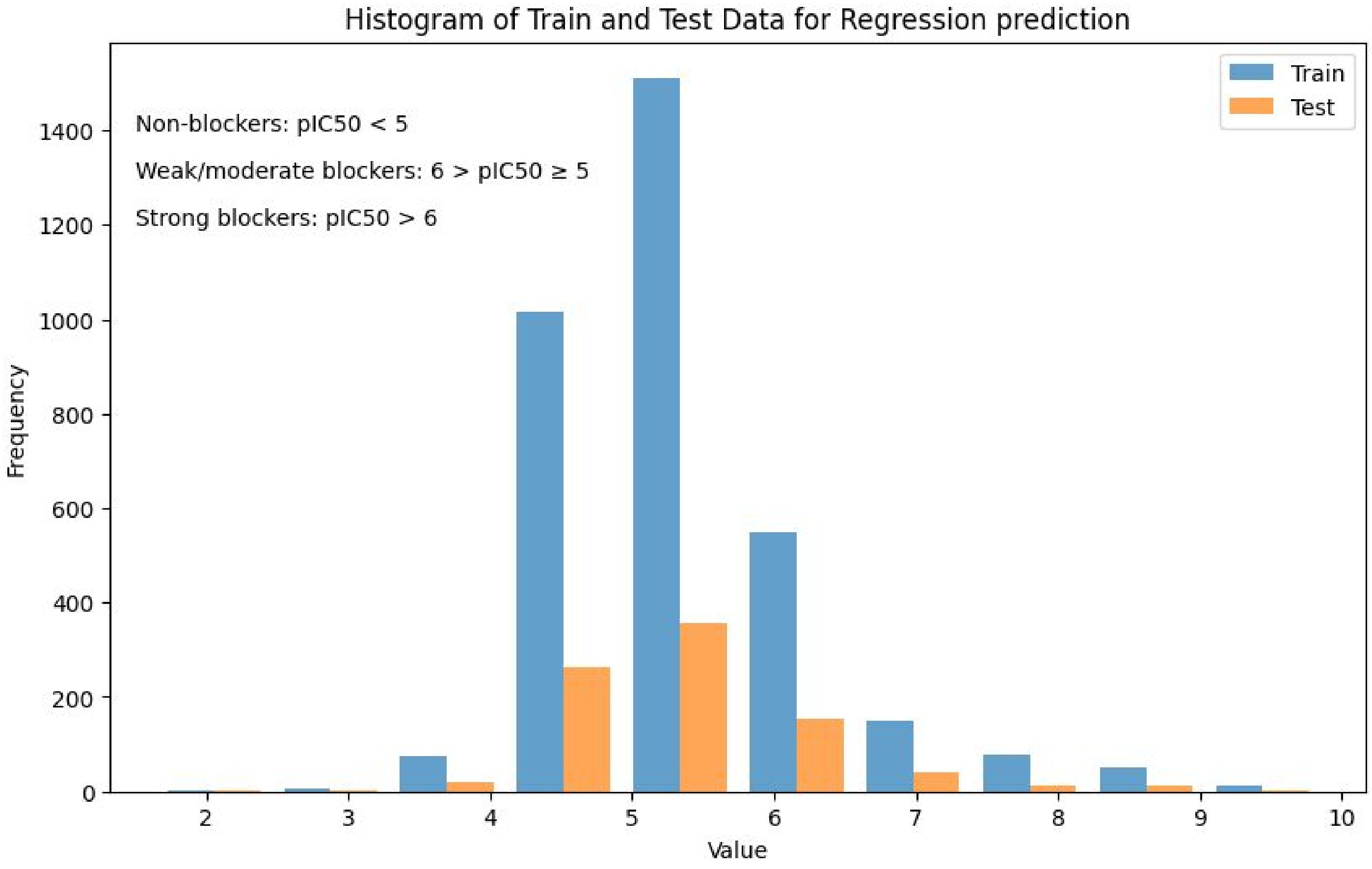

Figure S5 – Visualization of chemical space analysis using t-SNE for the comparison between the test set, training set, and benchmark datasets.

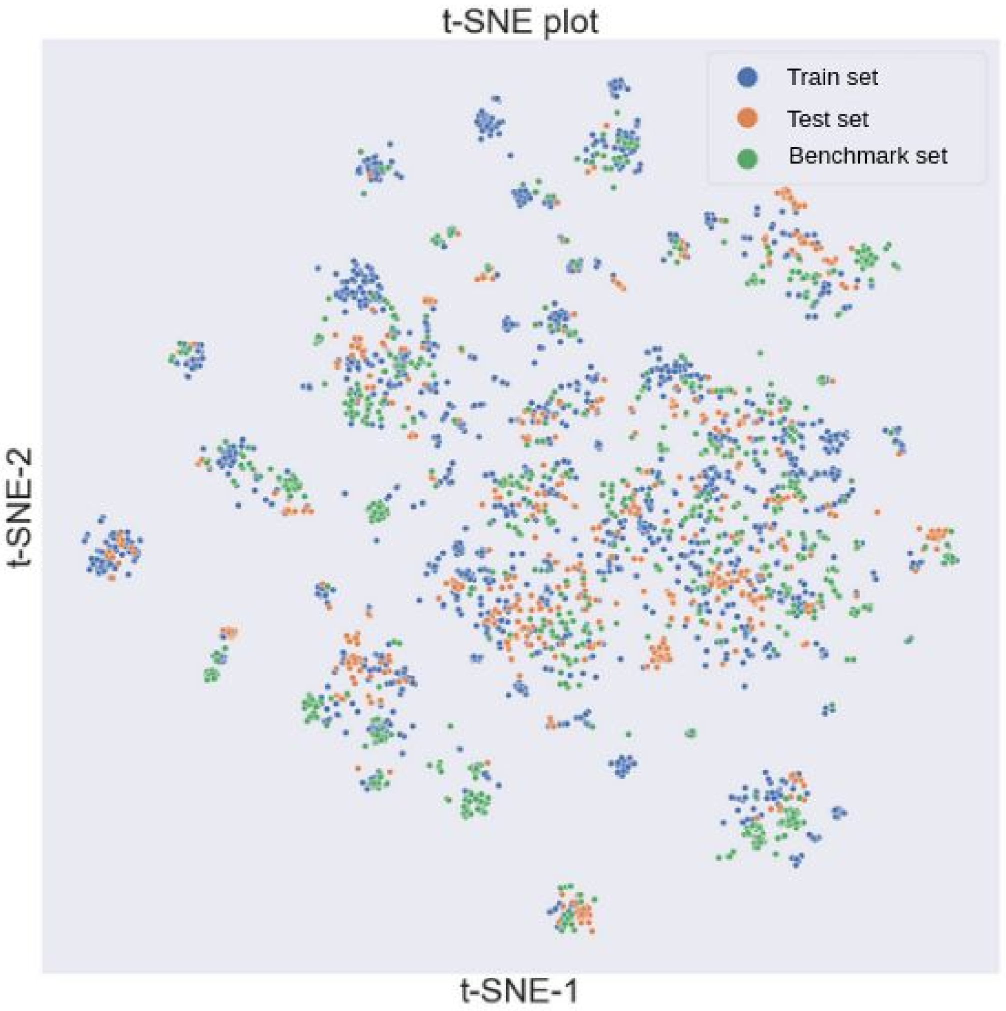

References

(1) Chemi, G.; Gemma, S.; Campiani, G.; Brogi, S.; Butini, S.; Brindisi, M. Computational Tool for Fast in Silico Evaluation of HERG K(+) Channel Affinity. Front Chem 2017, 5, 7. https://doi.org/10.3389/fchem.2017.00007.

(2) Wacker, S.; Noskov, S. Y. Performance of Machine Learning Algorithms for Qualitative and Quantitative Prediction Drug Blockade of HERG1 Channel. *Comput Toxicol* **2018**, *6*, 55–63. https://doi.org/10.1016/j.comtox.2017.05.001.

(3) Arab, I.; Wonneberger, D.; Barakat, K. ToxTree: Descriptor-Based Machine Learning Models to Predict HERG and Nav1. 5 Cardiotoxicity. **2022**.

(4) Siramshetty, V. B.; Chen, Q.; Devarakonda, P.; Preissner, R. The Catch-22 of Predicting HERG Blockade Using Publicly Accessible Bioactivity Data. *J Chem Inf Model* **2018**, *58* (6), 1224–1233. https://doi.org/10.1021/acs.jcim.8b00150.

(5) Karim, A.; Lee, M.; Balle, T.; Sattar, A. CardioTox Net: A Robust Predictor for HERG Channel Blockade Based on Deep Learning Meta-Feature Ensembles. *J Cheminform* **2021**, *13* (1), 60. https://doi.org/10.1186/s13321-021-00541-z.

(6) Creanza, T. M.; Delre, P.; Ancona, N.; Lentini, G.; Saviano, M.; Mangiatordi, G. F. Structure-Based Prediction of HERG-Related Cardiotoxicity: A Benchmark Study. *J Chem Inf Model* **2021**, *61* (9), 4758–4770. https://doi.org/10.1021/acs.jcim.1c00744.

(7) Sato, T.; Yuki, H.; Honma, T. Quantitative Prediction of HERG Inhibitory Activities Using Support Vector Regression and the Integrated HERG Dataset in AMED Cardiotoxicity Database. *Chem-Bio Informatics Journal* **2021**, *21*, 70–80.

(8) Sekhar Pagadala, N. Computational Prediction of HERG Blockers Using Homology Modelling, Molecular Docking and QuaSAR Studies. *Results Chem* **2021**, *3*. https://doi.org/10.1016/j.rechem.2021.100101.

(9) Meng, J.; Zhang, L.; Wang, L.; Li, S.; Xie, D.; Zhang, Y.; Liu, H. TSSF-HERG: A Machine-Learning-Based HERG Potassium Channel-Specific Scoring Function for Chemical Cardiotoxicity Prediction. *Toxicology* **2021**, *464*, 153018. https://doi.org/10.1016/j.tox.2021.153018.

(10) Wang, Y.; Huang, L.; Jiang, S.; Wang, Y.; Zou, J.; Fu, H.; Yang, S. Capsule Networks Showed Excellent Performance in the Classification of HERG Blockers/Nonblockers. *Front Pharmacol* **2019**, *10*, 1631. https://doi.org/10.3389/fphar.2019.01631.

(11) Ryu, J. Y.; Lee, M. Y.; Lee, J. H.; Lee, B. H.; Oh, K. S. DeepHIT: A Deep Learning Framework for Prediction of HERG-Induced Cardiotoxicity. *Bioinformatics* **2020**, *36* (10), 3049–3055. https://doi.org/10.1093/bioinformatics/btaa075.

(12) Munawar, S.; Windley, M. J.; Tse, E. G.; Todd, M. H.; Hill, A. P.; Vandenberg, J. I.; Jabeen, I. Experimentally Validated Pharmacoinformatics Approach to Predict HERG Inhibition Potential of New Chemical Entities. *Front Pharmacol* **2018**, *9*, 1035. https://doi.org/10.3389/fphar.2018.01035.

(13) Siramshetty, V. B.; Nguyen, D. T.; Martinez, N. J.; Southall, N. T.; Simeonov, A.; Zakharov, A. V. Critical Assessment of Artificial Intelligence Methods for Prediction of HERG Channel Inhibition in the "Big Data" Era. *J Chem Inf Model* **2020**, *60* (12), 6007–6019. https://doi.org/10.1021/acs.jcim.0c00884.
